# Barriers and facilitators to antenatal and delivery care in western Kenya: a qualitative study

**DOI:** 10.1186/s12884-015-0453-z

**Published:** 2015-02-13

**Authors:** Linda Mason, Stephanie Dellicour, Feiko Ter Kuile, Peter Ouma, Penny Phillips-Howard, Florence Were, Kayla Laserson, Meghna Desai

**Affiliations:** Child and Reproductive Health, Liverpool School of Tropical Medicine, Merseyside, UK; Kenya Medical Research Institute (KEMRI), Center for Global Health Research (CGHR), Kisumu, Kenya; Centers for Disease Control and Prevention (CDC), Atlanta, USA

**Keywords:** Qualitative, Antenatal care, Delivery care, Barriers, Facilitators, Western Kenya

## Abstract

**Background:**

In western Kenya, maternal mortality is a major public health problem estimated at 730/100,000 live births, higher than the Kenyan national average of 488/100,000 women. Many women do not attend antenatal care (ANC) in the first trimester, half do not receive 4 ANC visits. A high proportion use traditional birth attendants (TBA) for delivery and 1 in five deliver unassisted. The present study was carried out to ascertain why women do not fully utilise health facility ANC and delivery services.

**Methods:**

A qualitative study using 8 focus group discussions each consisting of 8–10 women, aged 15–49 years. Thematic analysis identified the main barriers and facilitators to health facility based ANC and delivery.

**Results:**

Attending health facility for ANC was viewed positively. Three elements of care were important; testing for disease including HIV, checking the position of the foetus, and receiving injections and / or medications. Receiving a bed net and obtaining a registration card were also valuable. Four barriers to attending a health facility for ANC were evident; attitudes of clinic staff, long clinic waiting times, HIV testing and cost, although not all women felt the cost was prohibitive being worth it for the health of the child. Most women preferred to deliver in a health facility due to better management of complications. However cost was a barrier, and a reason to visit a TBA because of flexible payment. Other barriers were unpredictable labour and transport, staff attitudes and husbands’ preference.

**Conclusions:**

Our findings suggest that women in western Kenya are amenable to ANC and would be willing and even prefer to deliver in a healthcare facility, if it were affordable and accessible to them. However for this to happen there needs to be investment in health promotion, and transport, as well as reducing or removing all fees associated with antenatal and delivery care. Yet creating demand for service will need to go alongside investment in antenatal services at organisational, staffing and facility level in order to meet both current and future increase in demand.

## Background

Despite a worldwide focus on the need to improve maternal health, maternal mortality and morbidity continues to be a significant problem in low – income countries. Maternal mortality and morbidity culminating from health, social, cultural and political inequalities, can be reduced by provision and utilisation of antenatal and emergency obstetric care (EmOC). Globally ~80% of maternal deaths are caused by complications during pregnancy, delivery or in the early postpartum period (haemorrhage, sepsis, unsafe abortion or pre/eclampsia), and 20% are due to diseases such as malaria and AIDS. It has been estimated that maternal mortality could be reduced by between 16-33% if there were a skilled birth attendant (SBA; i.e. doctors, nurses and midwives) at all deliveries to manage or treat these complications, although they should work within an ‘enabling’ environment, that is with sufficient and appropriate equipment, and are either able to manage an emergency or to refer on [1,2].

Whilst recent improvements have shown global mortality reduced by 47% from 1990-2010 [3], current estimates suggest that the maternal mortality rate remains high at 500 maternal deaths per 100,000 live births across Sub-Saharan Africa. Kenya typifies this lack of progress with an estimated maternal mortality ratio of 488 maternal deaths per 100,000 live births in 2008–9 [4] a figure higher than the reported 414 deaths per 100,000 live births in 2003 [5].In western Kenya where the current study was undertaken, estimates obtained from a Health and Demographic Surveillance System (HDSS) suggest 740 maternal deaths occur per 100,000 live births between 2003 and 2008 [6] significantly greater than the Kenyan national average.

Kenya currently has a 6 tier health care system, the basic unit is the community unit staffed by community health workers. Levels 2 and 3 are dispensary and health centres which provide preventive and curative care including health services for childbirth. The top 3 layers are higher level hospitals which focus more on curative and rehabilitation. Kenya offers focused antenatal care for all pregnant women to provide an integrated care package. This includes identification and management of obstetric complications and infections (including HIV and the prevention of mother to child transmission (PMTCT), syphilis and other sexually transmitted infections) as well as provision of prophylaxis for malaria through intermittent preventive treatment (IPTp) with Sulfadoxine- Pyrimethamine (SP), anaemia through provision of iron and folate and tetanus toxoid vaccination. ANC services are free in theory, but in practice most facilities require some payment for registration and for the recommended laboratory tests to be performed during the 1st visit as part of the ANC profile. As such, fees vary across facilities. HIV tests and prophylactic medications are offered free of charge.

The Kenyan MoH endorsed free delivery services in health centres and dispensaries in 2006. However women are commonly asked to pay a small fee (USD 1–2) for basic delivery supplies (such as gloves, cotton wool, sanitary maternity pads etc.). Government hospitals are allowed to charge a fee (usually ~ USD 6.50) but there is a waiver system for those who cannot afford this cost.

WHO recommends 4 visits for focused antenatal care (FANC), with the first visit scheduled in the first trimester [7,8]. One study conducted in western Kenya reported that 90% visited the antenatal clinic (ANC) at least once during their last pregnancy, although for 64% the first visit was not until the third trimester. Most women (64%) first visited the ANC in the third trimester. Furthermore studies also report that a high proportion of women still use the services of a traditional birth attendant (TBA) rather than a SBA. One study, again in western Kenya reported 80% of women delivered outside a health facility; among these, traditional birth attendants assisted 42%, laypersons assisted 36%, while 22% received no assistance [9]. A more recent study, again in western Kenya reported that 48% of births occurred in a health facility and 52% in a ‘non-institutional’ location [10].

Much research, particularly quantitative has been undertaken around why women fail to attend for ANC or do not deliver at health facility with the assistance of a SBA. Distance, cost and quality of care are well documented as major obstacles in the decision to seek obstetric care [11], however, a variety of additional factors shape women’s decisions if and when to seek care which are best explored using qualitative methods. In a recent systematic review, Gabrysch et al. [12] reported that despite modelling of co-variates to explain use of delivery care, there was still ‘*significant unexplained community level variation’* which could be explained by measurement error or ‘*omission of hard to measure factors’*. This would suggest that further work is needed in the local context, considering the economic, social, geographical and cultural specifics of the setting. The present study aims to look at why some women access antenatal or delivery care in formal health facilities in the western Kenya context whilst many do not.

## Methods

The study, which conforms to RATS guidelines, consisted of a series of 8 focus group discussions (FGDs) carried out in September 2010 as part of the formative research undertaken to understand and inform the best approach to setting up a prospective pharmacovigilance pregnancy cohort. The aim of this cohort was to monitor the safety of medications used during pregnancy which required understanding of the socio-cultural context and health seeking behaviour during pregnancy. Prior to beginning the research, community mobilisation took place involving a series of meetings held with DMOH, the chiefs, district officers and councillors, the community advisory board (CAB) set up by KEMRI/CDC and community members to introduce and get feedback on the proposed study plans. The results presented here look specifically at issues relating to choice of ANC and health facility based delivery care. The 2 research questions were 1) what are the barriers and 2) what are the facilitators to utilising ANC or delivery care within a health facility? FGDs were chosen to in order to gather the perspectives of a substantial number of local women, allowing for natural group dynamics to emphasise consensus and contradictions. It was felt that the topics were not too sensitive for group discussion. The interview guides covered themes relating to pregnancy recognition, disclosure and pregnancy related behaviour, pregnancy outcome, perception of adverse outcomes and practices around delivery. Due to time constraints, 4 FGDs concentrated mainly on issues around pregnancy, whilst the last 4 prioritised issues around delivery.

### Study area

The study was carried out in the rural area of Asembo in Rarieda district, western Kenya. With a midyear population of 64,442, recent estimates of maternal mortality ratio in this area are between 524 and 847 per 100,000 live births for the period 2003–2008 whilst infant mortality ranges from 76 – 132 per 1000 live births over the same period [13]. HIV and malaria rates are high, and anaemia is also a common problem. The population are predominantly members of the Luo tribe who are subsistence farmers or fishermen. Within the study area there are 7 health facilities (3 health centres, 3 dispensaries and 1 mission hospital) all of whom offer antenatal care. Women have to travel a maximum distance of 5 km to reach any facility.

### Recruitment and participants

Participants fitting the requirement for each group (see Table 1) were identified with the help of village reporters, using purposive sampling and invited to participate in the study. Village reporters are community based casual workers employed by KEMRI/CDC Research and Public Health Collaboration mainly to assist with community mobilisation for new studies and to act as community liaisons. The composition of the groups was intended to incorporate the various demographic groups of mothers within Asembo. Each group consisted of 8–10 women, aged from 15–49 years. The FGD’s were held at different community locations within Asembo (schools, village halls) so that women from neighbouring villages were able to attend. Each participant received reimbursement for transport. All FGDs were conducted in Dholuo and lasted approximately 1 hour and 45 minutes. Following oral informed consent which detailed all aspects of study participation including confidentiality within the group discussion, and anonymity of study participants outside the group context, all discussions were tape-recorded. Additional notes were taken to capture the main points. Both the moderator and the note taker were fluent in Dholuo and English. The discussions were transcribed verbatim and then translated from Dholuo into English. All transcripts were reviewed for accuracy by the moderator. The number of groups was decided upon at the outset and once data was collected, it was felt that this was sufficient to capture the range of views and as such data saturation had been reached.

**Table 1:** **Description of focus groups**

	**Participants**	**Number**
FGD1	Women of childbearing age (15–49) (WOCBA)	10 (from 4 villages)
FGD2	Recently or currently Pregnant women (RCPW)	8 (from 5 villages)
FGD3	Women of childbearing years (15–49) (WOCBA)	10 (from 4 villages)
FGD4	Adolescents (15–18 years) (Adolescents)	9 (from 2 villages)
FGD5	Adolescents (15–18 years) (Adolescents)	9 (from 2 villages)
FGD6	Recently or currently Pregnant women (RCPW)	9 (from 3 villages)
FGD7	Mothers of child born with an abnormality (MCBA)	9 (from 4 villages)
FGD8	Women of childbearing years (15–49) (WOCBA)	9 (from 3 villages)

### Ethics

The study was approved by the Kenya Medical Research Institute (KEMRI), the U.S. Centers for Disease Control and Prevention (CDC) and the Liverpool School of Tropical Medicine.

### Analysis

Thematic analysis was used to identify a narrow range of themes reflecting the textual data [14], using the following steps: The English transcripts were read several times by 2 of the authors (LM and SD) in order to familiarise themselves with the raw data. Neither author had been involved in data collection and considered themselves to be impartial in relation to study expectations. Each transcript was then entered onto Nvivo 9 where an initial skeleton coding frame was set up using the key themes identified by the authors. The coding frame comprised the major issues identified as barriers or facilitators to utilising either ANC or delivery care. Each transcript was then coded separately by the 2 authors according to the framework. On completion, they each revisited the framework and divided further using emergent subthemes. The narratives were then recoded where relevant, under these subthemes. The 2 separate completed coding frames were then compared to check consistency across them. Only minor differences were noted, which were discussed and agreement reached. The data for each theme and subtheme were then pieced together by one of the authors (LM) to provide an overview of the content relating to that specific theme. Quotes were chosen to represent a typical response, unless where stated, to illustrate a deviant opinion.

## Results

### Facilitators for health facility based antenatal care

Attending a health facility for ANC was viewed very positively by women across all of the FGDs with many reasons put forward as to why. Three elements of care seemed to be most important and raised by all groups: testing for disease, checking the position of the foetus, and receiving injections and/or medications.

In most cases, the women were vague in terms of the type of diseases they would be tested for, merely stating comments such as ‘*you have your blood tested so it is known whether you have a disease’* or referring to a check on the amount of blood*.* Malaria and HIV were the only diseases named. The women understood that it was important to know their HIV status in order to be treated and also to prevent passing the infection to their child. However, HIV testing was also seen as a barrier to attending for care.

‘*When we go to the clinic we are happy because our blood is tested so we know our status and this is something good that a TBA would not do’ P7 3 WOCBA*

Checking the position of the foetus was also a reason to access ANC – although some women acknowledged the TBA also performed this task so would visit them instead. Women reasoned this would determine whether their birth would be straightforward, or thought that the doctor would then be able to turn a malpresentation around.

*‘whether it is the child that is in a bad position, the doctor is there, you will leave that place when you have been helped totally’ P3 3 WOCBA*

Receiving injections was also deemed an important reason for clinic attendance. Although tetanus was mentioned specifically on occasion, very few women stated what the injection was for. The following illustrates the vague knowledge women possessed.

*‘I think they help us because when we get to clinic you get some injection. The injection would help the life of the child and me’ P8 2 RCPW*

Being provided medications was also proffered as a reason to attend ANC, although again, the purpose of being given medications was only vaguely understood i.e. ‘*to prevent disease’,’ to give energy’,’ to prevent infection’.* This was also considered to be helpful if a pregnant woman did not have ‘*enough’* blood. Overall, women appeared to trust and value the services provided at ANC.

‘*So when leaving the house in the morning it is clear in the mind that you will get some injections, some drugs and advice. So I would do whatever has been told to do. Those are the benefits of going to the clinic’ P7 2 RCPW*

Some women mentioned being given a registration card was the main motivation to attend ANC so that they could deliver in hospital in case of an emergency.

‘*She would rather go when she is left with a few days to delivery just for the purpose of securing a card so that even if things are difficult, she is not chased away’* P4 1 WOCBA

A few women also appreciated being given a free bed net.

*‘The services are good because say I am given a net, I will use it with my whole family. Secondly, my husband benefits too P7 3 WOCBA*

### Barriers for health facility based antenatal care

Four barriers to attending a health facility for ANC were predominant, mentioned by many of the women across the different groups. These were HIV testing, attitudes of clinic staff, which was linked to another barrier – long clinic waiting times, and cost.

Although some women felt it important to know their status, participants also spoke of others’ (rather than their own) fear of finding out their HIV status. This appeared to be partly a fear of not wanting to know their own status, or having others find out that they were HIV positive.

*‘And there are some people who if they hear about VCT they would rather die even if they are positive. They would die without taking the drugs’ P7 2 RCPW*

The attitudes of nursing staff also appeared to be a major barrier to attending ANC clinic.

‘*If you tell her that you have given birth to eight kids she would quarrel you and ask you if you still intend to give birth to more children. And maybe some of the children that she is talking about some had died. So you come from the clinic upset.’ P7 2 RCPW*

They were also deemed to have an unprofessional attitude, preferring to chat amongst themselves rather than working, which contributed to their habit of keeping the women waiting. Much criticism was levelled at long clinic waiting times, which was weighed up against the other duties that women had to attend to, a significant reason for non repeat attendance at clinic.

*‘you can stay in the [ANC] clinic for maybe two hours while critically sick and there is nobody to attend to you. When the nurse comes she would be harsh. So those are the problems we experience’ P2 2 RCPW*

This view was not however held by all; a couple of women when asked specifically, refuted this, reporting that they received care in good time, and did not spend a lengthy time at clinic.

Cost of ANC services was also a barrier preventing some women from attending ANC, either at all, or for repeat visits. However, not all women felt the cost, usually stated at US$ 0.12-0.23 per visit was unreasonable for its’ purpose. One group stated that

*‘Some people say the payment made is meant for the health of the child’ (p not recorded) 5 Adolescents*

However, costs mounted up particularly if tests were needed, and this had the effect of either preventing women from attending ANC at all, or making them visit as late as possible in their pregnancy so that they only needed to attend just once to check that there were no problems. This was also mentioned as a reason to visit a TBA whom the women can pay in commodities or in instalments.

*‘What I would say about the cost is that at times I am already 3 months pregnant and should be going to the clinic and I am supposed to pay 20/-, how I am going to get this 20/- I will not be able because at times I have children and that is what they are going to feed on so I am going to keep postponing that I will go next time if I get, yet there is no any other day that you will get it ready.’ P6 3 WOCBA*

A couple of women mentioned transport costs as an issue, and there was disagreement within groups as to whether the health facilities were far, although any distance was recognised as a problem when the women were sick and had to walk.

### Facilitators for health facility based delivery

Members of the groups were asked where their preference was for delivery. The overwhelming response was ‘*the hospital’* with women across the groups echoing this answer. The preference for a health facility delivery was largely due to the understanding that if complications occurred either during the delivery or in the postpartum period, this was the only place where they could be managed. Mostly just ‘complications’ were referred to, although specifically mentioned were excessive bleeding, retained placenta and having a large or badly positioned child which would require an operation.

*‘Sometimes the child might be big and if the TBA do not know how she would help then it would be difficult. So it is good if we give birth at the hospital because there are some children who are too big and requires the caesarean or if not complete caesarean then some part has to be cut to enable the baby to come out’. P7 7 MCBA*

Just one participant mentioned being HIV positive as a reason for a health facility delivery.

However, not all women agreed on health facility as the best place to deliver – it was suggested that a healthy woman would go to a TBA, ‘*although a sick mother would deliver in the hospital’.* A minority preferred to give birth at home (or at an older relative’s home) although sometimes this was simply for financial reasons, whilst just occasionally the view was expressed that it did not really matter where delivery took place – it was Gods will as to the outcome.

### Barriers to health facility based delivery

Although almost all participants agreed that health facilities were the safest place to deliver, a range of factors influencing the place of delivery and limiting women’s use of SBA were identified. These included: access to health facilities, which was influenced by unpredictable timing of labour and transport, as well as cost of facility-based delivery care, husbands’ preference, health facility staff attitudes and previous experiences and habits.

Distance to health facilities and lack of transport was cited in all FGDs as obstacles to delivering at a health facility. The timing and the unpredictability of the onset of labour, combined with distance to health facilities, played a critical role in determining where women deliver. This was the most common reason provided for preferring to deliver with a TBA.

*‘It depends, if the hospital is close then you go to the hospital [to deliver] and if the TBA’s places are close then it is the TBA’ P6 5 Adolescents*

*‘Getting a means at night to take you to the hospital may be a bit difficult; this will make you to go to a TBA’ p7 8 WOCBA*

The women’s preference for a health facility delivery was not in complete agreement with their own perceptions of men’s preference. Whilst there were women who thought that men also preferred their wives to attend health facilities for delivery, others disagreed, with cost being cited as the reason. One other viewpoint, although expressed by only a couple of women, was that the men do not care what their wives do at this time. The 3 contrasting viewpoints are:

*‘The men too want the hospitals because they hear that some women die while giving birth so they want the hospital’ P5 5 Adolescents*

*‘Sometimes the labour pain may begin when you are with your spouse and you tell him to accompany you to the hospital since you can’t walk on foot. He will respond that he is busy and moreover he doesn’t have money to take you to the hospital. This will force you to deliver at home because even if you go to the hospital he says that he warned you not to go there because he has no money’. P2 8WOCBA*

*‘There are some men who care, but very few’ P1 8 WOCBA*

Irrespective of preference, cost appeared to be the major barrier to attending a health facility for delivery.

*‘I know the hospital is the best it is only the lack of money.’P3 6 RCPW*

When asked if free services would make a difference to attendance for delivery most participants were very definitive, typically echoing:

*We would go’ P9 8 WOCBA*

*Yes, all people will go ‘P8*

*All people will go’ P4*

Cost and mode of payment were also mentioned as reason to deliver with TBA. Their services were perceived as affordable, friendly, and easily accessible at any time of day or night. Their fees are negotiable, relatively low, on average US$ 4 to 5 for delivery, though some offer services free of charge, or accept in-kind payments. A few participants, however, reported that TBAs can charge higher fees, as high as US$ 10 to 13 which is not dissimilar to the average fee charged at health facilities for normal delivery.

*‘I would go to a TBA because I know her so I wouldn’t have to struggle the huge hospital bill; I would only give her chicken and she would help me’ p4 8 WOCBA*

From all FGDs it was suggested that although TBAs had better interpersonal skills and were more accessible than health facilities, they had serious limitations such as not having adequate equipment for “safe delivery” such as gloves or medicines for PMTCT of HIV and were not able to help in case of obstructed labour when a caesarean section is required.

As with ANC, nurse attitudes were a barrier to health facility delivery, although to a lesser extent with fewer women mentioning this as a problem.

## Discussion

This paper presents findings from 8 focus group discussions carried out across Asembo in western Kenya. The discussions included both adolescents and women of childbearing age yet we were struck by the similar themes and opinions across the group discussions. This suggests that the opinions of the participants were not influenced by any member pressure which can occur during focus group discussions. Having a moderator that originated from the study area was also thought to reduce any response bias. Consequently we feel that the opinions of the participants were reliably obtained.

### Predicting readiness to act using a theoretical framework

We have used the “Health Belief Model” to assess our study participants ‘readiness to act’ in terms of attending a health facility for ANC and delivering within a health care setting [15]. The model was developed to explain behaviours but is also useful for health care programming in designing change strategies. The health belief model suggests that health-related behaviour depends on the individuals perception of four concepts:1. the severity of a potential illness, 2. the person's susceptibility to that illness, 3. the benefits of taking a preventive action 4. the barriers to taking that action. Positive implications for the future use of facility based antenatal and delivery care in the local area emerged from our study suggesting that change is possible, although strategies are needed to activate ‘readiness’ (cues to action). Women suggested that ANC services offered would be beneficial for their health and that of their baby. Perceived benefits of ANC were healthier pregnancy, healthier infant and a registration card or a bed net. Cost of care, nurses’ attitudes, long waiting times and HIV testing were perceived barriers to attending clinic. Most, though not all perceived susceptibility to complications, and understood the benefits of delivering in a health facility as complications would be managed there, however, as Thaddeus and Maine reported as far back as 1994 [11] cost was a substantial perceived barrier to delivery in a health facility. Importantly, results from our study indicate that many women are receptive, in theory, to using formal ANC and delivering within a healthcare facility. Cues to action are now needed in order to ensure these women are captured within the healthcare system that provides a continuum of care

### Failing to meet WHO recommendations

The present study provides explanations as to why most women in this area do not present at an ANC in their first trimester or attend the recommended 4 FANC visits [9]. Our results suggest that women wait until near the end of their pregnancy rather than first or even second trimester so that they have more time to gather the money to pay for any tests, also waiting until the last minute to get the card to present in case they had a health facility delivery. This also means that they do not have the time to attend for the recommended number of visits. Women were mostly positive about receiving ANC, recognising the importance of having a health check and receiving medications. However, once ascertained that they and the baby were fine, and having received a registration card, and/or a bed net cost, waiting time or nurses attitude were a greater barrier, following prior bad experience, or the reluctance to spend additional time away from home duties or additional cost, once the first visit had been made. This finding resonates with a recent systematic review [16] which reported that some women do not return to ANC once they have received their free ITN.

### Lack of knowledge negated by trust

We were not able to ascertain whether women were aware of the need for 4 visits but speculate this is unlikely because their knowledge appeared limited. Whilst mentioning the importance of receiving medications or injections none appeared to fully understand what these were for, an issue reported elsewhere [17,18], whilst a recent systematic review [16] found women were unaware of the benefits of intermittent preventive treatment (IPTp) with sulphadoxine-pyrimethamine (SP) for malaria, or why SP was being given. Vague knowledge of the services offered at ANC was also corroborated in a household cross-sectional survey carried out in the KEMRI/CDC HDSS in 2010 by Ouma P, Were V, Hamel M, Desai M. Results from the IPTp Uptake Cross-Sectional Survey [Unpublished]. KEMRI/CDC. 2010 ( [19]unpublished data). Women who were recently pregnant in the last 2 years were asked about information received at ANC. Just 56% reported that nurses providing care explained what they were doing. Despite this, women’s acceptance of medication appeared to demonstrate trust in both the facility based health care system, and in western medicine. Despite not knowing what they were for, participants took them believing they would help their, or the baby’s health. Likewise, Essendi et al. [19] reported women in Nairobi trusted health personnel implicitly, whilst a further study in Mombasa [20] similarly reported that women used and preferred western pharmaceuticals during their pregnancy. These findings contrast with other studies that show a distrust for modern medicine, and a stronger preference for traditional medicines and carers [21,22]. The study area has a high prevalence of malaria, also HIV, anaemia and helminth infections [13,23] necessitating screening during pregnancy. Malaria and HIV were the only infections/diseases mentioned often by pregnant women in our FGDs, and tetanus immunisation was mentioned just once. Increasing knowledge of services offered at ANC and their benefit may help earlier ANC attendance and compliance with the recommended minimum number of visits.

### Issues of male support

Women perceived their husbands provided little support, either financially or in terms of help with household duties, during pregnancy and delivery. This finding contrasts with the results from our FGDs among men, which focussed on the perspectives of men on antenatal and delivery care service utilisation in the same region [24]. In this parallel study, men were positive in their views of antenatal and delivery care, and as decision makers they stated they encouraged, some even ‘forced’, their wives to attend health facility for antenatal or delivery care. Further work would be useful to ascertain the true influence that men have on their spouses healthcare.

### The influence of healthcare provider attitudes

Our findings on service dissatisfaction contradict the result from the cross-sectional survey by Ouma P, Were V, Hamel M, Desai M. Results from the IPTp Uptake Cross-Sectional Survey [Unpublished]. KEMRI/CDC 2010 [19] (unpublished data), which showed 90% of respondents felt welcomed to ANC by the nursing staff. Furthermore, 71% felt they were encouraged to ask questions and 97% had them answered satisfactorily. In contrast, participants from our FGDs reported that the attitude of the nurses, as well as long clinic delays had the effect of preventing return visits. The difference in findings could be explained by the different methodological approaches used, the individual face to face survey being more prone to courtesy bias [25] compared to FGDs where participants are encouraged to share their feelings freely amongst their peers. Complaints about poor staff attitude has also been reported in studies in other low and middle income countries [26,27] and specifically in Kenya [20,28,29]. Common complaints about health care providers are lack of care, rudeness or harshness of attitude. Whilst nursing is a vocation, some may be attracted by the prestige associated with working in the health sector. Ojwang et al. [28] described nurses as ‘all knowing benefactor’, and the perception that some nurses feel superior or ‘*so learned’* was made by some women in our study. A poor attitude to patients by healthcare workers has long been recognised as a problem in the Kenyan setting, with the Kenya Ministry of Health declaring commitment to redress this issue. However, a study carried out in Kisumu, western Kenya [29] highlighted that the reduction in numbers of staff, coupled with increased workload led to burnout, which health workers admitted led to impatience and irritation with clients. There was some suggestion that nurses attitude or work ethic contributed to the long waiting times that were mentioned by many as a barrier to accessing ANC, however we are unable to assess whether this is a major factor, or whether this is primarily due to lack of organisation whereby women can turn up to clinic at any point during their pregnancy. Numbers can’t be predicted, neither can the nature of their individual care meaning health care professionals may have to perform a variety of roles in any session. This may be compounded by a shortage in healthcare workers in the Kenya health system which has been reported elsewhere [30-32]. Clearly however, investment is needed in staff numbers, alongside a shift in attitude of the healthcare workers with a parallel shift in the negative perceptions of women towards health care workers.

### HIV testing – barrier and facilitator

Studies [33,34] have demonstrated that HIV testing is a barrier to attendance at ANC, resulting from a fear of being tested because of involuntary disclosure to others, fear of stigma, and fear of finding out that they have the disease. These reasons were similarly important in our study, although participants also reported HIV testing as a reason for attending for care. It is likely that knowledge and use of ARVs and PMCT will mean that HIV testing becomes less of a barrier and more of a facilitator to antenatal services in the future. Indeed, Hardon et al. [35] in a 4 country study conducted reported PMCT services were valued because they protect the health of the unborn child and provide treatment for the mother. Kinuthia et al. [36] reported stigma was less of a barrier to using PMTCT than health service related issues.

### Cost – a significant barrier

As reported in numerous studies [16,19,37,38], cost was a barrier to ANC attendance, and to delivery in a formal healthcare setting. However, unlike other Kenyan settings where additional factors such as women’s lack of autonomy [19,27,39], HIV testing [33,37], and baby theft [37] including insecurity at night [19] had a strong negative influence, cost and transport were the predominant barriers arising through our FGDs. Women were aware that in cases where pregnancy complications arose, the healthcare setting was really the only place where they could deliver safely. What prevented them from doing this, was predominantly the cost. However, Ouma’s cross sectional survey which reported on actual behaviours, found the dominant reason for not delivering in a health care facility was that the labour/delivery came too fast. This was similarly reported in a recent Ghanaian study, and although 94% gave birth at home, only one quarter planned to do so [40].The timing of the onset and the unpredictability of labour was also mentioned as a barrier to deliver at a health facility in our FGDs. Individualized birth planning and preparedness is an integral part of FANC. However its implementation in Kenya has been poor, estimated at 68% in 2010 [41]. Full implementation of individualized birth planning could improve rates of delivery with a SBA as shown by Mpebemeni et al. [42]. Subsidizing cost of both transport and delivery fees are also likely to act as facilitators to delivery care. However, whilst these solutions might help create more demand for ANC and delivery with a SBA this also has to be balanced against inadequate supply. Currently, in western Kenya there are inadequate numbers of facilities able to provide quality delivery services, and a shortage of equipment and supplies for obstetric care [43].

### Study limitations

Some limitations need to be considered. Whilst our study included a number of focus groups, with women across a large age range our findings can be generalised only to the parent population, and not across other settings where different health care, geographical or cultural factors may affect access and utilisation of ANC and delivery services. It is possible, as with all focus group discussions in general, that the opinions given do not reflect each individual but rather the collective norm of the wider group. So whilst there appeared to be consensus around many issues within groups this may not reflect the true picture. However, similar opinions across groups reassure us that we have captured at least the prevailing views of women in Asembo. Furthermore, whilst it is possible there was an element of social desirability bias in response, with participant awareness that formal healthcare provision is being promoted by the government in preference to informal healthcare provided by the TBA, we uncovered opinion that disagreed the hospital is the best place in which to deliver. This suggests that some of the women felt comfortable in proffering opposing opinion. Constraints on time meant we were unable to explore some issues in depth, which may have added further value to the study. This includes exploring what the women understood by complications and their knowledge and recognition of danger signs, both during pregnancy and during labour. It would be useful to investigate whether they understand the need for planning ahead and what prevents any plans being put into practice successfully. Further understanding of the causes and possible solutions of long delays in ANC could help to address one of the main barriers to repeat ANC care.

## Conclusion

Our findings suggest that most women are amenable to ANC and would be willing and even prefer to deliver in a health facility, if it were affordable and accessible to them. However for this to happen there needs to be a range of enablers including health promotion for both women and also their partners in order that all fully understand the importance of attending for ANC and planning ahead in order facilitate delivery with a SBA. Further enablers which may help in womens’ decisions to use healthcare facilities include investment into nurse training in order to increase numbers and amend attitudes, and reducing or removing all fees. Subsidizing transport could further increase access to health facility based ANC and delivery care. However, we are mindful that if we create demand for services, there are inadequate numbers of trained staff and facilities able to provide quality ANC and delivery services, and certainly a shortage of equipment and supplies for emergency obstetric care. Both demand and supply factors need to be addressed in order to reduce the high maternal morbidity and mortality rate in the region.
